# Prevalence of Microbial Isolates Cultured from Endometrial Swab Samples Collected from United Kingdom Thoroughbred Mares from 2014 to 2020

**DOI:** 10.3390/vetsci11020082

**Published:** 2024-02-09

**Authors:** Rebecca Mouncey, Juan Carlos Arango-Sabogal, Polly Rathbone, Camilla J. Scott, Amanda M. de Mestre

**Affiliations:** 1 Department of Pathobiology and Population Sciences, The Royal Veterinary College, University of London, Hertfordshire AL9 7TA, UK; juan.carlos.arango.sabogal@umontreal.ca; 2 Département de Pathologie et Microbiologie, Faculté de Médecine Vétérinaire, Université de Montréal, Saint-Hyacinthe, QC J2S 2M2, Canada; 3 Department of Comparative Biomedical Sciences, The Royal Veterinary College, University of London, Hertfordshire AL9 7TA, UK; 4 Rossdales Veterinary Surgeons, Beaufort Cottage Stables, Newmarket CB8 8JS, UK; camilla.scott@rossdales.com; 5 Baker Institute for Animal Health, College of Veterinary Medicine, Cornell University, Ithaca, NY 14850, USA

**Keywords:** culture, cytology, endometrial, mare, Thoroughbred, prevalence

## Abstract

**Simple Summary:**

There is a lack of understanding as to whether the bacteria isolated from swabs of the surface of the lining of broodmares’ uteri (endometrial swabs) are present due to infection or contamination, or whether they are, in fact, part of the normal flora. This study aimed to understand this further by investigating endometrial swabs taken from a population of Thoroughbred broodmares and submitted to a laboratory in the UK between 2014 and 2020; by describing bacterial prevalence (the estimated percentage of swabs from which bacteria are isolated); and by evaluating whether bacterial prevalence changed over time or between mares of different ages and with differing amounts of inflammation of the uterine lining. Over the 7-year period, 18,996 endometrial swabs were submitted from 6050 mares on 290 farms. The overall prevalence of bacteria was 35.5%. Beta-hemolytic *Streptococcus* (17.9%) and *E. coli* (10.3%) were the most prevalent types of bacteria. The prevalence of bacteria changed over time (between different years in which mares were sampled) and was also impacted by the mare age and by whether there was inflammation of the uterine lining at the time of sampling. Results from this study provide up-to-date prevalence estimates and suggest that the interpretation of Thoroughbred endometrial swab findings is complicated.

**Abstract:**

Determining whether endometrial microbial isolates are pathogens, contaminants, or even part of the “normal” microbiome is extremely complex, particularly given the absence of “gold standard” tests for endometritis. Population-level benchmarking and temporal monitoring can provide novel insights and a wider context to improve understanding. This study aimed to (i) estimate the prevalence of endometrial isolates from swabs of Thoroughbred broodmares in Newmarket, UK between 2014 and 2020; and (ii) evaluate the effects of year, mare age, and cytology findings on isolate prevalence. Generalised linear mixed models with a logit link, both null models and models using year of sampling, mare age, or cytology findings as predictors, were fitted to estimate isolate prevalence. Over the 7-year period, data were available from 18,996 endometrial-swab samples from 6050 mares on 290 premises. The overall isolate prevalence was 35.5% (95% confidence interval (CI) 33.0–37.9), and this varied significantly between years. The most prevalent isolates were β-hemolytic *Streptococcus* (17.9; 95% CI: 17–19) and *E. coli* (10.3%; 95% CI: 9.0–11.6). Isolate prevalence increased with mare age except for *E. coli* isolates, and with increasing category of cytology findings except for α-hemolytic *Streptococcus* isolates. The results provide novel estimates of isolate prevalence and highlight knowledge gaps around potential complexities in the interpretation of findings.

## 1. Introduction

Bacterial endometritis is considered to be an important cause of infertility in thoroughbred broodmares [1]. As such, endometrial swabs to identify this condition are frequently taken at post-foaling and pre-breeding examinations, and in cases of infertility or pregnancy loss. Alongside this practice in the UK, the Horserace Betting Levy Board codes of practice [2] stipulate that freedom from infection from the venereal pathogens *Taylorella equigenitalis* (contagious equine metritis organism; CEMO), *Klebsiella pneumoniae* (capsular types 1, 2 and 5), and *Pseudomonas aeruginosa* must be established in all mares bred by natural cover, via an endometrial swab taken in oestrus, prior to every covering. As a result, large numbers of endometrial-swab samples are submitted for microbial testing and cytological evaluation during the Thoroughbred breeding season.

The Thoroughbred breeding season is short, with a demand from producers to breed mares as early as possible to realise the increases in sales price and race earnings associated with early-born foals [3]. In addition, the declining number of Thoroughbred stallions standing in the UK has led to increases in the covering-book sizes of established sires [4]. It has been hypothesised that such pressures are important contributing factors in the ever-increasing usage of reproductive therapeutics in Thoroughbred breeding practices [5,6], and, in particular, in the apparent widespread usage of intra-uterine antimicrobial therapies [5,6].

Historically, post-covering antibiotics were utilised due to concerns that endometrial inflammation observed at post-breeding examination occurred due to bacterial contamination of the uterus at covering [1]. In studies of endometrial samples from other geographical regions [7,8,9,10,11,12,13,14,15], *E. coli* and β-hemolytic *Streptococcus* were generally the most frequently described isolates; however, isolate frequency has varied widely between studies, depending on both the study population and sampling methodology. Given that it is now recognised that there is a complex bacterial microbiome present within both the equine uterus [16] and vagina [17], and that post-covering endometritis is a normal immunological response to insemination [1], the question of whether such isolates are pathogens, contaminants, or even “normal” commensals is extremely complex.

With a limited window for intra-uterine treatment once it has been determined that the post-breeding endometritis is persistent and therefore abnormal [1], and with the absence of a “gold standard test” to determine which mares truly require antimicrobial treatment [18], clinicians in the field are faced with important barriers to antimicrobial stewardship. Given these challenges and the current knowledge gaps around uterine “health” and “disease” in Thoroughbred breeding populations, population-level approaches can provide novel insights and a wider context to our understanding of pathogens found in the uterus that are candidates for causing disease. In the context of equine endometritis, benchmarking of the population-level prevalence of bacterial isolates, evaluation of the temporal changes in isolate patterns, and investigation of farm-, mare-, and swab-level factors associated with isolate prevalence are important first steps in furthering understanding towards informing best practice. The main objective of this study was, therefore, to estimate the prevalence of isolates cultured from all endometrial-swab samples from Thoroughbred mares submitted over 7 years (2014–2020) to a diagnostic laboratory in Newmarket, UK. For the most frequently cultured isolates, we also evaluated the effects of day-to-day variation and of mare and premises characteristics on the probability of isolating a given pathogen, and the associations between year of sampling, mare age, and endometrial cytology results and isolate prevalence.

## 2. Materials and Methods

This study forms part of a larger body of work that has also described antimicrobial resistance patterns in a subset of these isolates [19] and evaluated current diagnostic strategies [18].

### 2.1. Source Population, Study Population and Study Sample

**Figure 1:**
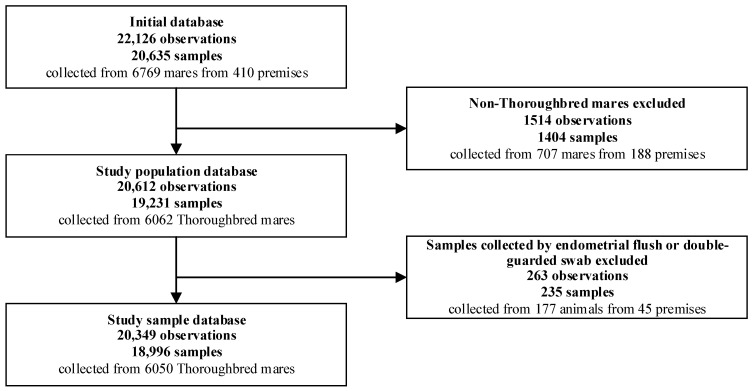
Number of observations in the initial database and excluded observations, and the study sample analysed to determine the prevalence of endometrial isolates cultured from swabs collected routinely from Thoroughbred mares between 2014 and 2020 in the United Kingdom. An observation corresponded to an isolate (when microbial growth was observed) or a negative result (when no microbial growth was observed).

The source population included 22,126 observations obtained from 20,636 endometrial samples collected from 6769 mares, from 410 premises, between 2014 and 2020 (Figure 1). An observation corresponded to an isolate when microbial growth was observed, or as a negative result when microbial growth was not observed. Therefore, where the growth of a mixed culture was detected, multiple observations could have originated from the same sample. Observations from non-Thoroughbred mares were excluded from the initial database (1514 observations corresponding to 1404 samples collected from 707 mares, from 188 premises). Observations from samples collected by endometrial flush and double-guarded swabs were excluded (263 observations corresponding to 235 samples collected from 177 mares, from 45 premises). Finally, the study sample included 20,349 observations obtained from 18,996 endometrial-swab samples collected from 6050 mares, from 290 premises.

### 2.2. Sample Collection and Laboratory Analyses

Endometrial swabs were collected during oestrus prior to natural cover or for diagnostic sampling post-mating or post-foaling (e.g., in cases of suspected endometritis or pregnancy failure). Following aseptic preparation of the perinium, a sterile single-use speculum was inserted into the mare’s vagina, through which two unguarded swabs were inserted sequentially into the uterus via the cervix. The first swab was then placed into charcoal medium for microbial culture and the second swab was placed into a plain sheath with no medium for cytology. Swabs were transported unrefrigerated to Rossdales Laboratory, Newmarket (UK). At the laboratory, samples from charcoal medium were plated, and samples with no transport medium smeared, within 12 h of collection.

#### 2.2.1. Microbial Cultures

Swabs were plated onto Blood Agar and MacConkey Agar for aerobic culture, as well as onto Chocolate Blood Agar for microaerophilic culture (5–10% CO_2_). All plates were kept at 37 °C and checked after 12–24 h then again at 48 h. Colonies were identified using the Analytical Profile Index system (bioMérieux, Craponne, France). Microbial isolates were reported and included in this study when at least one colony was identified.

#### 2.2.2. Cytology

For the cytology, endometrial smears were stained with Pollacks Rapid Trichrome stain on a Leica Autostainer XL (Lecia Biosystems, Milton Keynes, UK). The whole slide was scanned, and results were averaged then graded based on the percentage of polymorphonuclear neutrophil (PMN) cells in the endometrial cell sample in each high power field as follows: 0 (No PMN); +/− (≤0.5% PMN); 1+ (>0.5–5% PMN); 2+ (>5–30% PMN); and 3+ (>30% PMN).

### 2.3. Data Analyses

Initially, a description of the study sample was performed, including the number of samples, mares, and farms by year, the distribution of the ages of the mares. The numbers of samples submitted and the numbers and proportions of samples with microbial growth were described both overall and stratified by year of sampling. Then, the overall frequencies of all isolated microorganisms were described. Alongside this, the distributions of the numbers of culture-positive swabs, cytological findings, and number of species isolated, and the distributions of the most commonly isolated species, and the number of species isolated were described.

#### 2.3.1. Outcomes and Explanatory Variables

The outcome of interest was whether an isolate could be cultured at a given sampling visit, and this was only assessed for the most frequently isolated microorganisms. There were no explanatory variables measured at the premises- and mare-levels. At the observation level, explanatory variables included a continuous variable for the age of the mare at the time of sampling, and categorical variables for the year of the visit (from 2014 to 2020) and for the cytology results for the mare at that sampling visit.

#### 2.3.2. Prevalence of Endometrial Isolates

The prevalence of the most frequently isolated microorganisms were quantified using the marginal predicted mean probability estimated from a null (i.e., a model without fixed predictors) generalised linear mixed model (GLMM) with a logit link. The mean number of units at the upper level of clustering (i.e., isolates per sample, samples per mare, mares per premise) was calculated. Given that the mean number of observations per sample was 1.1 and the outcome was measured at the observation level, the sample level was omitted from the data-hierarchy structure [20]. Therefore, the GLMM (1) was built to account for the clustering of observations by mare and by premises as follows:

(1) $$\mathrm{Logit}(p_{ijk})=\beta_{0}+v_{\mathrm{premise}(k)}+u_{\mathrm{mare}(jk)}$$

where *p_ijk_* is the probability of the outcome for the *i*th observation (i.e., the observation made on a given sampling visit on a given mare) of the *j*th mare from the *k*th premises; *β*_0_ is the intercept; *v_premise(k)_* is the premises random effect, normally distributed with a mean of 0 and variance *σ_p_*^2^; and *u_mare(jk)_* is the mare random effect with a mean of 0 and variance *σ_m_*^2^.

##### Effects of Day-to-Day Variation and Mare- and Premises Characteristics on the Prevalence of Endometrial Isolates

Using the simulation method described previously [21] and SAS statistical software (v. 9.2, SAS Institute Inc., Cary, NC, USA), the variance obtained from the previous null GLMM was partitioned to explore the variation in the probability of the outcomes attributable to each of the data levels (observation/day-to-day; mare; and premises).

##### Prevalence of Endometrial Isolates by Year of Submission, Age of the Mare and Inflammatory Response

To estimate the prevalence of the most frequently cultured isolates by year, mare age, and category of inflammatory response, the following GLMM (2) was fitted for each outcome (i.e., “Any type of isolate”, β-hemolytic *Streptococcus*, *Escherichia coli*, Coagulase Negative *Staphylococcus*, *Staphylococcus aureus*, and α-hemolytic *Streptococcus*):

(2) $$\mathrm{Logit}\left(p_{ijk}\right)=\beta_{0}+\beta_{1}x_{ijk}+v_{\mathrm{premise}(k)}+u_{\mathrm{mare}(jk)}$$

where *p_ijk_* is the probability of the outcome for the *i*th observation of the *j*th mare from the *k*th premises; *β*_0_ is the intercept; *β*_1_ is a vector of regression coefficients for the explanatory variables “year of sampling”, “age of the mare”, and “cytology result”, which were included in the model one at a time; *v_premise(k)_* is the premises random effect; and *u_mare(jk)_* is the mare random effect, both assumed to be normally distributed and have a mean of 0 and variance of *σ_p_*^2^ and *σ_m_*^2^, respectively.

The assumption of linearity of the continuous explanatory variable “age of the mare” with the log odds of each outcome was assessed graphically. Mare age was categorised by quartile, given that the assumption of linearity was not met. An interaction between the variables “age of the mare” and “cytology” was explored by adding an interaction term between them into the model for each outcome. Interaction terms were only retained if *p* < 0.05. Whenever an interaction between age of the mare and cytology was observed (*p* < 0.05), results were then presented as proposed by [22] or graphically. The predicted probability of the outcome was estimated for each model using the margins command in Stata statistical software (v. 17; StataCorp LLC, College Station, TX, USA). The normality of residuals and their homoscedasticity at the highest level (premises) were assessed graphically using Q-Q plots and plots of residuals against the predicted values.

## 3. Results

### 3.1. Study Sample

The numbers of samples collected, mares sampled, and farms visited stratified by year are presented in Supplementary Figure S1. Mare age at the sampling visit varied from 2 to 25 years (mean = 9.6; median = 9).

Overall, microbial growth was observed from 5785 samples (30.5%; 95% CI: 29.8–31.1; Table 1). The numbers of samples submitted and the numbers and proportions of samples (and 95%CI) with microbial growth stratified by year is presented in Table 1. Although the number of samples submitted and the number of mares sampled were similar for all years, there was variation in the proportion of samples with growth between years.

The frequencies and relative frequencies, with 95% CIs, of all microorganism species isolated from the study population are presented in Table 2 below. In total, 33 different microorganism species were isolated, of which β-hemolytic *Streptococcus* (*n* = 3738; 52.4%; 95% CI: 51.2–53.5) and *E. coli* (*n* = 1842; 25.8%; 95% CI: 24.8–26.8) were the most frequent species (Table 2). *Taylorella equigenitalis* (CEMO) was not isolated from this population and the frequencies of *K. pneumoniae* and *P. aeruginosa* were very low (relative frequencies 0.6 and 0.4%, respectively; Table 2).

Of the 18,996 samples, 17,938 had a concomitant cytology result available (94.4%; Table 3). The distributions of cytology and culture findings and of number of isolates identified (mono or mixed cultures) are presented in Table 3. Proportions of samples with positive-microbial growth increased between cytology grades 0, +/−, 1+ and 2+; however, proportions were similar between grades 2+ and 3+. Proportions of mono- and mixed cultures did not vary between cytology grades.

The distribution of the number of isolates identified (number, proportion, and 95%CI) from the 5785 endometrial swabs from which at least one isolate was identified is presented in Table 4, with data stratified by four out of the five (as the Coagulase Negative *Staphylococcus* GLMM did not converge) most-prevalent isolates. Overall, in 79.1% (95%CI 79.1: 78.0–80.1; *n* = 4577) of the positive samples, only one microorganism species was isolated (monocultures). In the remaining 1208 samples, more than one microorganism species (mixed cultures) was isolated (two isolate species in 1071 samples; three isolate species in 129 samples; and four isolate species in 8 samples). The proportions of monocultures with β-hemolytic *Streptococcus* and *E. coli* isolates were higher than the proportions of monocultures with *S. aureus* and α-hemolytic *Streptococcus* isolates.

### 3.2. Prevalence of Endometrial Isolates

Five different outcomes occurred with sufficient frequency for the GLMM models to converge (“Any type of isolate”, β-hemolytic *Streptococcus*, *E. coli*, *S. aureus*, and α-hemolytic *Streptococcus*), and their prevalence are reported in Table 5. The GLMM for Coagulase Negative *Staphylococcus* did not converge; therefore, prevalence for this isolate will not be presented further. When accounting for clustering at the mare and premises levels, the overall prevalence of endometrial isolates (any type of isolate) was 35.5% (95% CI: 33.0–37.9; Table 5), and β-hemolytic *Streptococcus* (17.9%; 95% CI: 16.8–19.0) and *E. coli* (10.3%; 95% CI: 9.0–11.6) had the highest individual isolate prevalence Table 5). Isolate prevalence varied between β-hemolytic *Streptococcus*, *E. coli*, and *S. aureus*. However, the prevalence of *S. aureus* and α-hemolytic *Streptococcus* isolates were similar, but they were much lower than the other three isolates.

### 3.3. Effects of Day-to-Day Variation and Mare and Premises Characteristics on the Prevalence of Endometrial Isolates

The proportion of the prevalence explained by day-to-day (i.e., sampling visit), mare, and premises characteristics are presented in Table 5. Mare-level characteristics explained the largest proportion of the prevalence of any type of isolate (0.57), *E. coli* isolates (0.61), *S. aureus* isolates (0.75), and α-hemolytic *Streptococcus* isolates (0.95). On the other hand, the prevalence of β-hemolytic *Streptococcus* isolates was explained mainly by day-to-day/sampling visit-level characteristics (0.51).

### 3.4. Prevalence of Endometrial Isolates stratified by Year of Sampling, Mare Age, and Inflammatory Response

**Figure 2:**
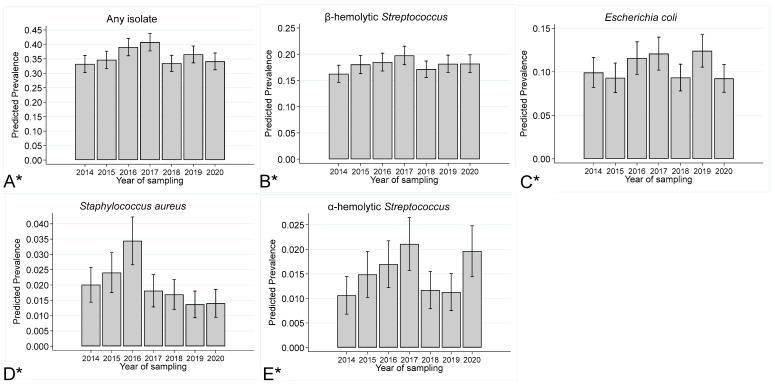
Prevalence (marginal predicted probabilities)^1^ and 95% confidence intervals stratified by year of sampling for the following: (**A**) any type of isolate, (**B**) β-hemolytic *Streptococcus*, (**C**) *E. coli*, (**D**) *Staphylococcus aureus*, and (**E**) α-hemolytic *Streptococcus* isolates cultured from endometrial swabs collected from Thoroughbred mares between 2014 and 2020 in the United Kingdom. ^1^ Estimates were obtained from a generalised linear mixed model incorporating 20,349 observations, from 6050 mares on 290 premises. *—*p* < 0.05.

The prevalence and 95% CIs by year of sampling for any type of isolate, β-hemolytic *Streptococcus* isolates, *E. coli* isolates, *S. aureus* isolates, and α-hemolytic *Streptococcus* isolates are presented in Figure 2 below, where it can be seen that significant between-year variation in isolate prevalence were observed. The temporal prevalence pattern of β-hemolytic *Streptococcus* isolates appeared to be similar to the temporal pattern of overall isolate prevalence; however, temporal patterns for *E. coli*, *S. aureus*, and α-hemolytic *Streptococcus* isolates appeared to vary.

**Figure 3:**
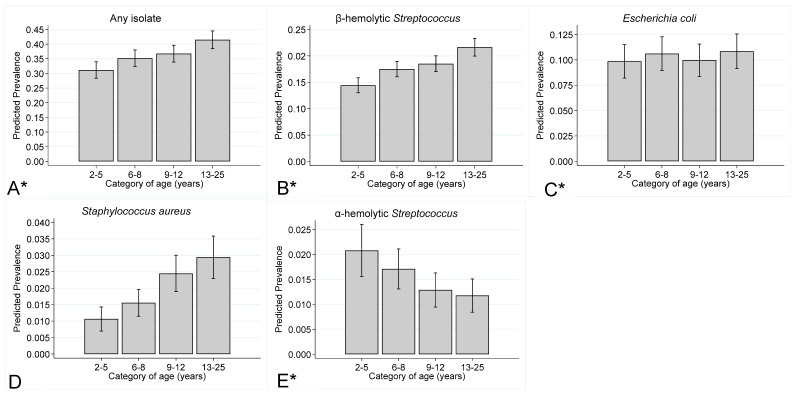
Prevalence (marginal predicted probabilities)^1^ and 95% confidence intervals, stratified by mare age for (**A**) any type of isolate, (**B**) β-hemolytic *Streptococcus*, (**C**) *E. coli*, (**D**) *Staphylococcus aureus*, and (**E**) α-hemolytic *Streptococcus* isolates cultured from endometrial swabs collected from Thoroughbred mares between 2014 and 2020 in the United Kingdom. Mare age was categorised using quartiles.^1^ Estimates were obtained from a generalised linear mixed model incorporating 20,349 observations, from 6050 mares on 290 premises. *—*p* < 0.05.

Figure 3 presents the prevalence of any type of isolate, β-hemolytic *Streptococcus* isolates, *E. coli* isolates, *Staphylococcus aureus* isolates, and α-hemolytic *Streptococcus* isolates by category (quartile) of mare age. The prevalence of any type of isolate, β-hemolytic *Streptococcus* isolates, and *Staphylococcus aureus* isolates increased with mare age, and the prevalence of α-hemolytic *Streptococcus* isolates decreased with mare age. The prevalence of *E. coli* isolates, however, was not associated with mare age.

**Figure 4:**
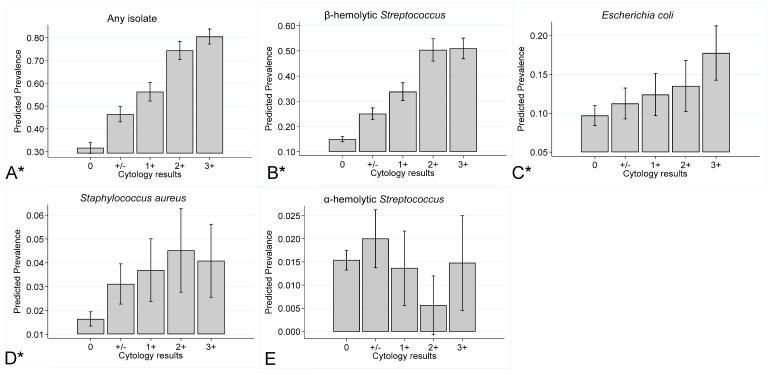
Prevalence (marginal predicted probabilities)^1^ and 95% confidence intervals, stratified by cytology result category for (**A**) any type of isolate, (**B**) β-hemolytic *Streptococcus*, (**C**) *E. coli*, (**D**) *Staphylococcus aureus*, and (**E**) α-hemolytic *Streptococcus* isolates cultured from endometrial swabs collected from Thoroughbred mares between 2014 and 2020 in the United Kingdom. Cytology results categories represent the percentage of polymorphonuclear neutrophils cells (PMN) per high power field: 0 (No PMN); +/− (≤0.5% PMN); 1+ (>0.5–5% PMN); 2+ (>5–30% PMN); and 3+ (>30% PMN).^1^ Estimates were obtained from a generalised linear mixed model incorporating 20,349 observations, from 6050 mares on 290 premises. *p* < 0.05.

Figure 4 presents the prevalence for any type of isolate, β-hemolytic *Streptococcus* isolates, *E. coli* isolates, *S. aureus* isolates, and α-hemolytic *Streptococcus* isolates stratified by mares’ cytology findings. The prevalence of any type of isolate, β-hemolytic *Streptococcus* isolates, *E. coli* isolates, and *S. aureus* isolates increased with the percentage of PMN. The prevalence of α-hemolytic *Streptococcus* isolates, however, was not associated with percentage of PMN.

**Figure 5:**
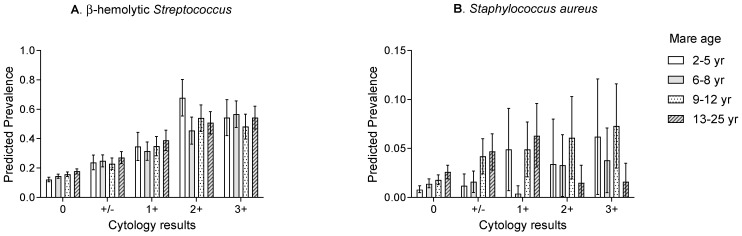
Prevalence (marginal predicted probabilities)^1^ and 95% confidence intervals of (**A**) β-hemolytic *Streptococcus* and (**B**) *Staphylococcus aureus* isolates grouped by cytology result category and mare age. Estimates were obtained from a generalised linear mixed model incorporating data of isolates cultured from endometrial swabs collected from Thoroughbred mares between 2014 and 2020 in the United Kingdom. ^1^ Estimates were obtained from a generalised linear mixed model incorporating 20,349 observations, from 6050 mares on 290 premises.

An interaction between mare age and cytology results was observed for β-hemolytic *Streptococcus* and *S. aureus* isolates, suggesting that the associations observed between the prevalence of these outcomes and mare age varied depending on the cytology results. These interactions are displayed graphically in Figure 5, where it can be seen that the prevalence of β-hemolytic *Streptococcus* and *S. aureus* increased with mare age only when no (and +/− in the case of *S. aureus*) PNM were observed on the cytology smear. No interactions between mare age and cytology results were observed for any of the remaining outcomes (any type of isolate, *E. coli* isolates, and α-hemolytic *Streptococcus*).

## 4. Discussion

We present here a comprehensive overview of the population-level prevalence of endometrial isolates from over 18,000 endometrial-swab samples from Thoroughbred broodmares in the UK. Collected over a 7-year period, these results provide novel benchmarking for the industry and highlight important areas for future research. Overall, isolates were identified in around a third of samples. Beta-hemolytic *Streptococcus* and *E. coli* were the most prevalent isolates. Temporal variation (between years) in prevalence was observed for all of the most prevalent isolates. Prevalence was associated with mares’ cytology findings, and β-hemolytic *Streptococcus* but not *E. coli* prevalence was associated with mare age. For β-hemolytic *Streptococcus* isolates, the majority of prevalence variation was observed at the swab-level (variation in prevalence between samples within mares). In contrast, the majority of variance in *E. coli* prevalence was observed at the mare level (variation in prevalence between mares). *T. equigenitalis* (CEMO) was not isolated from this population and *K. pneumoniae* and *P. aeruginosa* were rarely isolated.

The overall prevalence of all types of isolates reported in the present study (35%) is in keeping with isolate frequencies reported from a study of 2311 uterine swab samples submitted to a laboratory in Ithaca, USA between 2007 and 2017 (39%) [17]. It is also similar to those from 8269 pre-breeding or diagnostic uterine samples from 7655 mares (swabs (95%), flushes or biopsies) submitted to a laboratory in Florida, USA between 2003 and 2008 (31%) [18], and from 394 routine pre-breeding samples (315 swabs, 79 flushes) submitted to a laboratory in Italy between 2014 and 2018 (33%) [19]. It is, however, much higher than frequencies reported from studies exclusively using guarded swabs for 2660 routine pre-breeding samples collected from 1621 mares on 17 Thoroughbred studs in Australia (7%) [10] and 2123 pre-breeding and diagnostic samples collected from 970 mares in the USA between 2001 and 2004 (11%) [11]. It is also lower than those reported from uterine swabs of 363 mares with positive endometrial cytology/vaginal discharge and repetitive infertility from five studs in Spain (89%) [12], from studies of 4122 (89%) [13] and 586 (49%) [14] uterine swab samples from mares suffering with reproductive disorders in Italy, and from a study of 293 swabs from mares with fertility problems sent to a laboratory in Sweden (64%) over the 1996–1997 breeding season [15]. It is important to consider that, unlike in the present study, all of the other studies reported only the proportion of positive samples rather than prevalence estimates that accounted for any data clustering. Accounting for data clustering, for example, multiple swabs from one mare, allows for the longitudinal nature of the data and is vital in order to produce accurate parameter estimates [20], making the present findings important and novel epidemiological information for the industry. Despite this, current available evidence [7,8,9,10,11,12,13,14,15] suggests that isolate distribution appears to vary depending both on swabbing technique (guarded vs. unguarded) and the population sampled (routine versus clinical evidence of uterine infection). In the present study, mares’ clinical information was not available. However, given the UK’s codes of practice for Thoroughbred mares [2] and the fact that all samples submitted to the laboratory over the study period were included, it is reasonable to assume that a large proportion were routine pre-breeding samples.

In keeping with the present study, all of the previously discussed studies in which individual isolate frequencies were reported [7,8,10,11,12,13,14,15] consistently described β-hemolytic *Streptococcus* and *E. coli*, as the most frequent isolates, although in different proportions. In the present study, β-hemolytic *Streptococcus* and *E. coli* accounted for 52% and 26% of isolates. In the previously mentioned studies, frequencies of β-hemolytic *Streptococcus* ranged from 28–37% and *E. coli* from 17–39% of all isolates. In addition, a study from Italy [9], which used additional enrichment techniques for bacterial isolation, reported α-hemolytic *Streptococcus* spp. (27%) in addition to *E. coli* (27%) and β-hemolytic *Streptococcus* (26%), and the study of mares with endometritis in Spain [12] reported *Staphylococcus* spp. (15.6%) in addition to *E. coli* (17.3%), and *Streptococcus* spp. (13.5%) as the most frequent isolates. Compared to previous studies, the present study population appears to have less isolate diversity, with β-hemolytic *Streptococcus* or *E. coli* accounting for over three quarters of all isolates (78%). However, it must be considered that isolation methodologies varied between studies, particularly in terms of types of plate media and the use of enrichment; therefore, direct comparisons must be made with caution.

*Taylorella equigenitalis*, the causative organism of CEM, is notifiable in the UK, and the monitoring of temporal changes in isolate prevalence of this and other venereal pathogens, such as *K. pneumoniae* and *P. aeruginosa*, forms an important part of national infectious disease surveillance programmes [2]. Reassuringly, T. equigenitalis was not isolated, and *K. pneumoniae and P. aeruginosa* levels were extremely low in the present study population.

In the present work, temporal variation in isolate prevalence was observed for all isolates combined, and it was observed individually for the four most common isolates. Similar temporal variation in isolate frequency was also described in some of the previously mentioned studies [8,13]. Temporal variation could simply reflect differences in the sample population between years, for example in the present study; although the total number of samples submitted per year did not show much variation (Table 1), there may have been variation in the proportion of routine to diagnostic samples submitted to the laboratory. Temporal variation in isolate distribution can also be an indication of changes in AMR within a population [12]. There is growing concern over the risk of antimicrobial resistance (AMR) in the reproductive tract of broodmares, due to the widespread usage of antibiotics both in intra-uterine treatments and semen extenders [23]. As such, the AMR patterns of present isolates have also been reported [19], where it can be observed that, particularly for *E. coli* isolates but perhaps also for β-hemolytic *Streptococcus* isolates, the patterns of temporal variation in prevalence reported here appear to follow patterns of temporal variation in AMR [19].

It is recognised that, compared to younger mares, older broodmares are more likely to have poor conformation of the vulva and perineal region alongside defects in myometrial contractions, lymphatic drainage, mucociliary clearance, and cervical function, which can all predispose them to additional bacterial contamination of the reproductive tract and endometritis [24]. As such, the effects of mare age on the prevalence of endometrial isolates, alongside interactions between mare age and cytology results, were evaluated in the present work. As expected, overall isolate prevalence increased with mare age, along with the prevalence of β-hemolytic *Streptococcus* and *S. aureus* isolates. However, the prevalence of α-hemolytic *Streptococcus* decreased with mare age and *E. coli* prevalence was not associated with mare age in the present study population. In the context of these relationships, it is interesting to note the observed interaction between mare age and cytology findings for both β-hemolytic *Streptococcus* and *S. aureus* isolates. Most notably, that the prevalence of β-hemolytic *Streptococcus* and *S. aureus* increased with the mare age only when no or +/− PMN were observed on the cytology smear. This suggests, perhaps, that older mares, due to the mechanisms discussed above, may be predisposed to uterine contamination with β-hemolytic *Streptococcus* and *S. aureus* organisms during the sampling procedure, hence the absence of inflammation in these samples. It could also be considered that *S. aureus* was most frequently isolated alongside other organisms (70% of samples from which it was isolated), which perhaps adds further weight to a contamination hypothesis [10,11].

Variance partitioning by level was evaluated in the present study (Table 5), where β-hemolytic *Streptococcus* isolates were notably different when compared to the other most prevalent isolates. In the case of β-hemolytic *Streptococcus* isolates, the majority of variance (0.51) was observed at the sample/isolate level, suggesting that the presence of this isolate is mainly due to factors occurring at the time of sampling. Such factors could, therefore, include mare age at the time of sampling, the stringency of the sampling technique, and, of course, the status of the mare in terms of whether it was a routine pre-breeding sample or was from a clinical case of infertility or endometritis. Conversely, for *E. coli*, for example, the majority of variance (0.61) was observed at the mare level, suggesting that the presence of this isolate is mainly due to factors intrinsic to the individual mare, which are less likely to change between samples, i.e., over time. This is a finding that may also align with the previously discussed absence of association between *E. coli* prevalence and mare age. Intrinsic factors could, therefore, include the mares’ endometrial immune response, uterine clearance capacity, or perhaps even the constituents of her “normal” endometrial microbiome. In relation to this, it is interesting to observe that in a study of vaginal isolates from clinically normal mares, *E. coli* was the most common species, isolated from 23/26 mares prior to insemination [17], perhaps adding yet further complexity to the clinical interpretation of *E. coli* isolates, particularly in routine pre-breeding samples.

The question of whether the isolates from endometrial samples are pathogens, contaminants, or even part of the “normal” microbiome is extremely complex [16], particularly given the absence of “gold standard” tests for endometritis [18]. It must also be considered, as was the case in the present study, that the use of non-guarded swabs is likely to increase the risk of sample contamination. As isolate information must be interpreted in the light of the clinical findings of various examinations at the time of sampling, endometritis prevalence was not reported or estimated in this population. Historically, *E. coli* and β-hemolytic *Streptococcus* (*S. equi subsp. zooepidemicus)* have been associated with 50% to 80% of all bacterial endometritis cases in published data [25]. It is interesting to note that, in the present study, the majority (72% and 65%, respectively) of β-hemolytic *Streptococcus* and *E. coli* isolates were monocultures, which was in contrast to α-hemolytic *Streptococcus* and *S. aureus* (42% and 34% of isolates in monoculture, respectively). Across the previously mentioned studies, monocultures are proposed as being more likely to represent pathogenic isolates, given their association with reduced pregnancy and live-foaling rates [10,11]. Interestingly, in the previous studies, despite overall proportions of monocultures varying widely (35–93%) [9,10,11,12,13,14], the studies of mares with clinical endometritis or fertility issues report the highest proportion of monoculture isolates [12,13,14].

A key criterion often utilised for the diagnosis of endometrial infection is the presence of inflammation in combination with positive microbial culture [8]. This rationale is, however, not without potential flaws, as studies comparing sampling methods have found endometrial-swab samples to be particularly unreliable in terms of their ability to identify bacterial endometritis [8,26]. Alongside this, there is also evidence to suggest that not all potentially pathogenic bacteria are associated with an inflammatory response [27,28,29], with isolation of microorganisms from uterine swab samples in the absence of inflammation on cytological examination reported in several studies [11,26,30]. In the present work, isolates were identified in 26% of all samples that had 0 PMN. However, isolate prevalence was observed to increase significantly with inflammation grade on cytological examination, with the notable exception of α-hemolytic *Streptococcus* isolates. Such a lack of association between isolate prevalence and cytology findings has been reported in previous studies, but in contrary to our findings, this has been mainly observed with *E. coli* isolates [11,30,31].

Overall, just 19% of our samples for which cytology findings were available had any evidence of inflammation (PMN > 1), a proportion identical to that (19%) described in the study of 2123 guarded pre-breeding and clinical swab samples in America [11], despite the overall proportion of samples with isolates identified being considerably higher in our study (30% cf. 11%). It must, however, be acknowledged that inflammation cut-off points and cytology groupings were different between the two studies. Nevertheless, there is ongoing debate around the sensitivity and specificity of cytology and culture findings from endometrial swabs, where it is recognised that both false positives and negatives occur and vary depending on both the method used to collect the sample [30,32] and the chosen positive cut-off point [18]. Given that our isolate prevalence estimates are much higher than those from studies using guarded swabs, despite cytology findings being similar, it is quite possible that some isolates might, therefore, represent contaminants from the unguarded sampling technique utilised in this work. However, it is also interesting to note that the current proportions of monocultures did not appear to vary between categories of cytology results (Table 4). Therefore, it must also be considered that the observed discordant results between culture and cytology could also be due to the imperfect accuracy (sensitivity and specificity) of both tests. This was suggested in our previous work [18] using Bayesian latent class models, where regardless of the culture case definition, the sensitivity and specificity of cytology was even lower than previously reported for swab samples in studies using histology as the reference standard test. To demonstrate this in the present population (Table 3), at least half of the samples with PMN <5% (+/− and 1+) yielded no isolate growth, compared to just 20% of samples with PMN >5% (2+ and 3+). This could illustrate how tests may perform differently depending on the spectrum of “disease” [33]; for example, in the present population there appears to be more agreement (i.e., positive cytology and culture) in those samples with higher inflammatory grades. It is also possible that some swabs may have been taken at the foal heat, where the presence of PMN is part of the normal physiology of uterine involution and not necessarily associated with bacterial infection [10].

A study evaluating the effectiveness of endometrial cytology and culture in predicting live-foaling rates in a group of 1621 Thoroughbred mares in Australia found that a cytology of >1 PMN compared to 0 PMN, and/or any monoculture compared to a negative culture, were both associated with significantly lower live-foaling rates [10]. If these two thresholds associated with live-foaling rate were applied to our population-level study, between 19 and 24% of samples could theoretically be associated with reduced live-foal rates, a figure that is significantly maligned with the widespread use of post-covering treatments (estimated to be utilised in up to 73% mares) and intra-uterine antimicrobials (included in 50% of intra-uterine post-covering treatments) in Thoroughbred broodmares in the UK [5,6]. This, perhaps, brings into question the need for some of these interventions, and suggests that more judicial use of antimicrobials may be required [34,35], alongside the urgent need for work to explore such associations in a UK setting. It has been argued that, due to the short ~48-h window following cover to administer intra-uterine treatments, there may be cases where cytology and/or culture results would not be available in time to guide therapeutic decision making [1]. However, given the stipulation of UK Thoroughbred breeding codes for a negative endometrial swab result prior to covering [2], and the low reported prevalence of post-covering fluid accumulation and endometritis in Thoroughbred broodmares [6,36], this situation is likely in practice to be extremely uncommon. It is vital, therefore, that future efforts are focused towards providing scientific evidence that allows the achievement of consensus on the best practices in terms of sampling methods, diagnostic tests, and interpretation of findings, and towards identifying and understanding drivers of therapeutic decision making in the field.

As previously discussed, the main limitations of this work were the retrospective nature of the data and the lack of clinical information from the time of sampling. Post-foaling, routine pre-breeding, and diagnostic samples from clinical cases were all included; therefore, the data cannot be used to accurately estimate the prevalence of clinical endometritis. However, given that the study utilised population-level data and that analysis methods have been utilised to account for the clustered nature of the data, the current estimates provide important epidemiological information. In summary, this study has, for the first time, estimated the population-level prevalence of isolates in over 18,000 endometrial-swab samples collected over a 7-year period from Thoroughbred broodmares in the UK, and has evaluated the variation in overall prevalence and the prevalence of the most common isolates according to year of sampling, mare age and cytology findings. These results provide much-needed population-level benchmarking of key bacterial species in a UK setting and compliment the findings from our larger body of work [18,19], which aims to highlight important limitations in current practices in terms of the use of unguarded endometrial swabs and the routine pre-breeding swabbing of clinically normal mares. Our findings also highlight knowledge gaps in the understanding of uterine “health” and “disease” in Thoroughbred broodmares and can inform future research to improve both antimicrobial stewardship and Thoroughbred reproductive health and productivity.

## Figures and Tables

**Table 1:** Number and proportion (95% confidence intervals (CI)) of samples with microbial growth out of the number of endometrial swabs submitted and the number of mares sampled per year from a total of 6050 Thoroughbred mares between 2014 and 2020 in the United Kingdom.

Year of Sampling	Number of Samples Submitted	Number of Mares Sampled	Number of Samples with Microbial Growth	Percentage (95% CI) of Samples with Microbial Growth
2014	2621	1413	707	27.0 (25.3–28.7)
2015	2500	1365	725	29.0 (27.2–30.8)
2016	2684	1447	886	33.0 (31.2–34.8)
2017	2633	1444	919	34.9 (33.1–36.7)
2018	2936	1709	817	27.8 (26.2–29.5)
2019	2862	1593	901	31.5 (29.8–33.2)
2020	2760	1518	830	30.1 (28.4–31.8)
Total	18,996	6050	5785	30.5 (29.8–31.1)

**Table 2:** Frequencies and relative frequencies of microorganism species cultured from 18,996 endometrial swabs collected from 6050 Thoroughbred mares between 2014 and 2020 in the United Kingdom.

Microorganism Isolated	Frequency	Percentage	95% Confidence Interval
β-hemolytic *Streptococcus*	3738	52.4	(51.2–53.5)
*Escherichia coli*	1842	25.8	(24.8–26.8)
*Staphylococcus aureus*	441	6.2	(5.6–6.7)
Coagulase Negative *Staphylococcus*	391	5.5	(5.0–6.0)
α-hemolytic *Streptococcus*	308	4.3	(3.8–4.8)
*Actinobacillus species*	133	1.9	(1.6–2.2)
*Enterococcus faecalis*	50	0.7	(0.5–0.9)
*Klebsiella pneumoniae*	43	0.6	(0.4–0.8)
*Acinetobacter species*	29	0.4	(0.3–0.6)
*Pseudomonas aeruginosa*	27	0.4	(0.2–0.5)
*Bacillus species*	27	0.4	(0.2–0.5)
*Enterobacter aerogenes*	22	0.3	(0.2–0.4)
*Enterococcus species*	16	0.2	(0.1–0.3)
*Proteus species*	14	0.2	(0.1–0.3)
*Pasteurella species*	10	0.2	(0.0–0.2)
*Enterobacter species*	6	0.1	(0.0–0.2)
*Pantoea agglomerans*	6	0.1	(0.0–0.2)
*Pseudomonas fluorescens*	5	0.1	(0.0–0.2)
*Corynebacterium species*	4	0.1	(0.0–0.1)
*Enterobacter agglomerans*	4	0.1	(0.0–0.1)
Non-hemolytic *Streptococcus*	4	0.1	(0.0–0.1)
*Mucor species*	3	0.0	(0.0–0.1)
*Aspergillus fumigatus*	2	0.0	(0.0–0.1)
*Kluyvera species*	2	0.0	(0.0–0.1)
*Pseudomonas stutzeri*	2	0.0	(0.0–0.1)
*Achromobacter species*	1	0.0	(0.0–0.1)
*Burkholderia cepacian*	1	0.0	(0.0–0.1)
*Enterobacter cloacae*	1	0.0	(0.0–0.1)
Fungus	1	0.0	(0.0–0.1)
*Klebsiella oxytoca*	1	0.0	(0.0–0.1)
*Pseudomonas putida*	1	0.0	(0.0–0.1)
*Pseudomonas species*	1	0.0	(0.0–0.1)
*Weeksella virosa*	1	0.0	(0.0–0.1)
Total	7138	100%	-

**Table 3:** Distributions of cytology results, microbial growth, and number of isolates of 17,938 endometrial swabs collected from 5745 Thoroughbred mares between 2014 and 2020 in the United Kingdom. Cytology results are presented as the percentage of polymorphonuclear neutrophils cells (PMN) in the endometrial epithelial cell sample per high power field.

Cytology Result		Microbial Growth (%: 95% Confidence Interval)		Isolates (%: 95% Confidence Interval)	
	Number of Swabs	No	Yes	Monocultures (1 Isolate)	Mixed Cultures (>1 Isolate)
0 (No PMN)	14,440	10,707 (74.1: 73.4–74.9)	3733 (25.9: 25.1–26.6)	2941 (78.8: 77.4–80.1)	792 (21.2: 19.9–22.6)
+/− (≤0.5% PMN)	1829	1110 (60.7: 58.4–62.9)	719 (39.3: 37.1–41.6)	562 (78.2: 75.0–81.0)	157 (21.8: 19.0–25.1)
1+ (>0.5–5% PMN)	733	361 (49.3: 45.6–52.9)	372 (50.8:47.1–54.4)	295 (79.3: 74.9–83.1)	77 (20.7: 16.9–25.1)
2+ (>5–30% PMN)	462	127 (27.5: 23.6–31.7)	335 (72.5: 68.3–76.4)	268 (80.0: 75.4–83.9)	67 (20.0: 6.1–24.6)
3+ (>30% PMN)	474	104 (21.9: 18.4–25.9)	370 (78.1: 74.1–81.6)	305 (82.4: 78.2–86.0)	65 (17.6: 14.0–21.8)
Total	17,938	12,409 (69.2: 68.5–69.8)	5529 (30.8: 30.1–31.5)	4371 (79.1: 78.0–80.1)	1158 (20.9: 19.9–22.0)

**Table 4:** Distribution of the number of isolates identified from 5785 endometrial swabs from which at least one isolate was identified, collected from Thoroughbred mares between 2014 and 2020 in the United Kingdom.

Isolate	Total	Monoculture (%: 95% Confidence Interval)	Mixed Culture (%: 95% Confidence Interval)
Any type of isolate	5785	4577 (79.1: 78.0–80.1)	1208 (20.9: 19.8–21.9)
β-hemolytic *Streptococcus*	3738	2700 (72.2: 70.1–73.6)	1038 (27.8: 26.3–29.2)
*Escherichia coli*	1842	1192 (64.7: 62.5–66.9)	650 (35.3: 33.1–37.5)
*Staphylococcus aureus*	441	151 (34.2: 30.0–38.8)	290 (65.8: 61.2–70.0)
α-hemolytic *Streptococcus*	308	131 (42.5: 37.1–48.1)	177 (57.5: 51.9–62.9)

**Table 5:** Prevalence (marginal predicted probabilities) and variance partitioning ^1^ for the most frequent bacterial isolates, cultured from endometrial swabs collected from Thoroughbred mares between 2014 and 2020 in the United Kingdom.

Isolate	Marginal Predicted Probability (95% CI)	Variance Partitioning by Level		
		Farm	Mare	Sample/Isolate
Any type of isolate	35.5 (33.0–37.9)	0.26	0.57	0.17
β-hemolytic *Streptococcus*	17.9 (16.8–19.0)	0.17	0.32	0.51
*Escherichia coli*	10.3 (9.0–11.6)	0.29	0.61	0.10
*Staphylococcus aureus*	2.0 (1.6–2.3)	0.22	0.75	0.03
α-hemolytic *Streptococcus*	1.5 (1.3–1.7)	0.02	0.95	0.03

^1^ Estimates were obtained from a null generalised linear mixed model on 20,349 observations from 6050 mares on 290 premises.

## Data Availability

The original contributions presented in the study are included in the article/Supplementary Material, further inquiries can be directed to the corresponding author/s.

## Supplementary Materials

The following supporting information can be downloaded at: https://www.mdpi.com/article/10.3390/vetsci11020082/s1, Figure S1: Number of samples collected (A), mares sampled (B), and premises visited (C) per year to determine the prevalence of microbial isolates cultured from endometrial swabs collected routinely from Thoroughbred mares between 2014 and 2020 in the United Kingdom.
