# Supplementary material for: Prevalence of Microbial Isolates Cultured from Endometrial Swab Samples Collected from United Kingdom Thoroughbred Mares from 2014 to 2020
Source: Vet Sci. 2024 Feb 9;11(2):82. doi: 10.3390/vetsci11020082 (PMC10891641; doi:10.3390/vetsci11020082)
Supplement: Supplementary file 1 [file vetsci-11-00082-s001.zip › vetsci-2746160-supplementary.pdf]

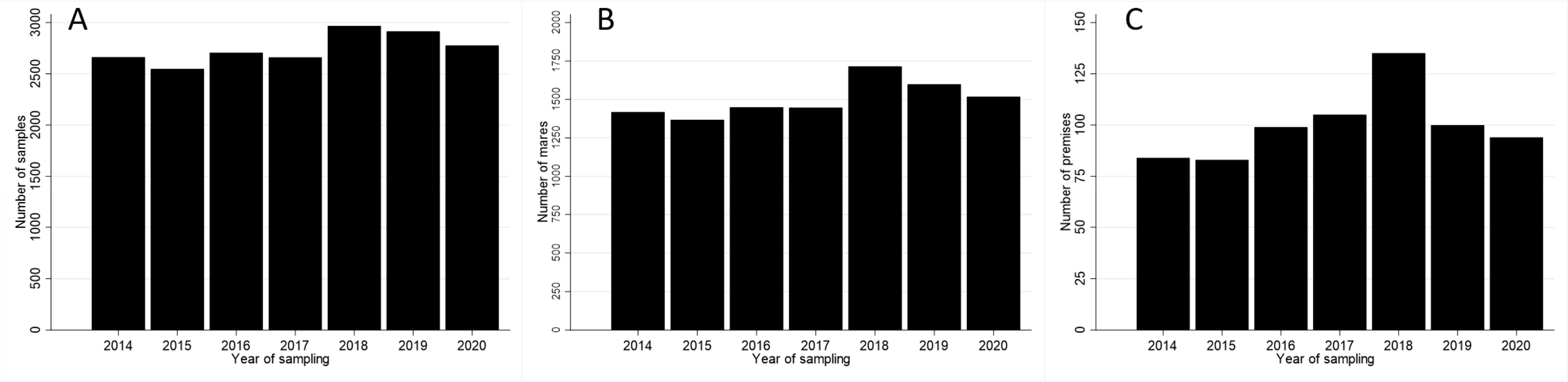

Figure S1. Number of samples collected (A), mares sampled (B), and premises visited (C) per year to determine the prevalence of microbial isolates cultured from endometrial swabs collected routinely from Thoroughbred mares between 2014 and 2020 in the United Kingdom.
